# Cancer patient perceptions on the ethical and legal issues related to biobanking

**DOI:** 10.1186/1755-8794-6-8

**Published:** 2013-03-08

**Authors:** Zubin Master, Jaime O Claudio, Christen Rachul, Jean CY Wang, Mark D Minden, Timothy Caulfield

**Affiliations:** 1 Alden March Bioethics Institute, Albany Medical College, 47 New Scotland Avenue, MC 153, Albany, NY, 12208-3478, USA; 2 Health Law and Science Policy Group, Rm 461 Law Centre, University of Alberta, 89 Avenue and 111 Street, Edmonton, AB, T6G 2H5, Canada; 3 Ontario Cancer Institute, Princess Margaret Hospital, University Health Network, 101 College Street, TMDT 3-912, Toronto, ON, M5G 1L7, Canada; 4 Campbell Family Cancer Research Institute/Ontario Cancer Institute, University Health Network, Faculty of Medicine, University of Toronto, MaRS TMDT 8-363 101 College Street, Toronto, ON, M5G 1L7, Canada; 5 Princess Margaret Cancer Center, Campbell Family Cancer Research Institute/Ontario Cancer Institute, University Health Network, Faculty of Medicine, University of Toronto, 610 University Avenue, 9-113, Toronto, ON, M5G 2M9, Canada; 6 Health Law and Science Policy Group, Rm 462 Faculty of Law and School of Public Health, Law Centre, University of Alberta, 89 Avenue and 111 Street, Edmonton, AB, T6G 2H5, Canada

**Keywords:** Biobank, Tissue repository, Cancer patient perspectives, Consent, Withdrawal, Anonymity, Incidental findings, Return of results, Ownership, Trust

## Abstract

**Background:**

Understanding the perception of patients on research ethics issues related to biobanking is important to enrich ethical discourse and help inform policy.

**Methods:**

We examined the views of leukemia patients undergoing treatment in clinics located in the Princess Margaret Hospital in Toronto, Ontario, Canada. An initial written survey was provided to 100 patients (64.1% response rate) followed by a follow-up survey (62.5% response rate) covering the topics of informed consent, withdrawal, anonymity, incidental findings and the return of results, ownership, and trust.

**Results:**

The majority (59.6%) preferred one-time consent, 30.3% desired a tiered consent approach that provides multiple options, and 10.1% preferred re-consent for future research. When asked different questions on re-consent, most (58%) reported that re-consent was a waste of time and money, but 51.7% indicated they would feel respected and involved if asked to re-consent. The majority of patients (62.2%) stated they had a right to withdraw their consent, but many changed their mind in the follow-up survey explaining that they should not have the right to withdraw consent. Nearly all of the patients (98%) desired being informed of incidental health findings and explained that the information was useful. Of these, 67.3% of patients preferred that researchers inform them and their doctors of the results. The majority of patients (62.2%) stated that the research institution owns the samples whereas 19.4% stated that the participants owned their samples. Patients had a great deal of trust in doctors, hospitals and government-funded university researchers, moderate levels of trust for provincial governments and industry-funded university researchers, and low levels of trust towards industry and insurance companies.

**Conclusions:**

Many cancer patients surveyed preferred a one-time consent although others desired some form of control. The majority of participants wanted a continuing right to withdraw consent and nearly all wanted to be informed of incidental findings related to their health. Patients had a great deal of trust in their medical professionals and publically-funded researchers as opposed to profit-based industries and insurance companies.

## Background

The large-scale collection of biological materials along with health and demographic information has become an important research tool in many areas of the biomedical sciences. Biobanking involves the collection and long-term storage of biological materials (e.g., blood, DNA, urine, cells, etc.) and health information. Biobanks are valuable research platforms that allow for future research studies on stored samples. Investigators can, for example, analyze data derived from biological samples (i.e., presence of a gene sequence), and correlate that analysis with other data (e.g., health information or demographics) to identify statistical relationships. Biobanking can also allow the sharing of biological samples with other researchers. Large-scale biorepositories have been viewed as necessary to improve our understanding of disease and to develop new diagnostic and treatment modalities. Substantial investments have been made at local, state, and national levels to create biobanks in jurisdictions such as the United Kingdom, United States, Sweden, Iceland, Canada, Denmark, Finland, and several other countries [1-4].

Biobanks have generated a range of ethical and legal challenges related to privacy, informed consent [5], control and ownership [6,7], withdrawal of samples and consent [8], commercialization, return of results and incidental findings [9], and research governance. Indeed, these issues have generated much policy debate and have already resulted in several public controversies regarding informed consent [10-12], commercialization [13], and control, and ownership [14-16]. Gaining a greater understanding of the perspectives of different stakeholders, including patients who provide biological samples and health information to biobanks can offer insight into the nature and drivers of these ethical controversies, inform policy development, and assist in public engagement.

While there are many studies evaluating public and patient perspectives on biobanking, only a few capture the views of Canadians [17-21] and even fewer capture those of patients. Because patients may be more invested in research and have different expectations than the general public [22], gaining an understanding of the views of this population will help to provide insight into the breadth of perspectives relevant to biobanking. In this study, we performed a survey of leukemia patients receiving treatment at Princess Margaret Hospital’s Hematology Oncology Clinic on several issues related to biobanking, including informed consent, withdrawal, anonymity, return of results, ownership, privacy, and trust. Patients were provided with written surveys during an initial visit and the same survey was provided six months later in order to determine whether their views had changed on various ethical and legal issues regarding biorepository research. A six month period provided sufficient time for patients to receive treatment and interact with physicians and clinic staff, which may have influenced their views about biobanking and participation in research.

## Methods

### Research setting

As part of a large research study designed to understand the perspectives of different Canadian stakeholder groups on biobanking, we recruited 100 patients from June 22 to October 12, 2011 who received treatment for leukemia at six different hematology-oncology clinics at Princess Margaret Hospital in Toronto, Ontario, Canada. Patients were receiving chemotherapy or other treatment for leukemia and some were in remission. As two of the authors (MM and JW) are co-directors of the Princess Margaret Hospital Hematologic Malignancy Tissue Bank, patients participating in this bank were invited to participate in our survey as a convenience sample. All patients were 16 years of age or older, and the majority lived in the Greater Toronto Area and surrounding municipalities although a few lived in Northern Ontario. The Greater Toronto Area has a multicultural and multiethnic population.

Patients were informed by their attending hematologist that a researcher (JC) might approach them to participate in the biobank survey. A short consent form explaining the study was provided along with a verbal explanation. After consent was obtained, patients were left with the survey to complete and a copy of the informed consent form. This project received research ethics approval from the University of Alberta Research Ethics Board (MS2_Pro00021037) and the University Health Network Research Ethics Board (11-0380-BE) prior to patient enrolment.

A total of 100 out of 156 patients participated by completing the initial survey providing a response rate of 64.1%. Patients were given ample time to fill out the survey and most were completed while the patient was still in the clinic; two patients completed the questionnaire at home and returned it by mail. Seventy eight patients that participated in the survey had already consented for their samples to be deposited in the Princess Margaret Hospital Hematologic Malignancy Tissue Bank (REB 01-0573-C) at the time the survey was given to them. At the time of recruitment for the initial survey, patients were also asked if they would agree to participate in a follow-up survey six months later. Of the 72 patients who provided consent to complete a follow-up survey, only 45 returned a completed follow-up survey giving a response rate of 62.5%. Of the 100 patients participating in the initial survey, 60 were male and 40 were female. For the follow-up survey, 27 males and 18 females participated thereby maintaining the 60–40 male to female ratio in both the initial and follow-up surveys.

### Survey design and analysis

This survey is part of a larger initiative designed to understand and compare the opinions of different stakeholders, including the public [22], on a breath of research ethics topics related to biobanking. Survey questions were designed based on (1) a systematic search of the published academic literature in order to identify the ethical and legal challenges associated with biobanking [23], and (2) an analysis of specific surveys on the ethical and legal issues associated with biobanking. Interviewers at the University of Alberta’s Population Research Laboratory pre-tested the survey with 20 Albertans from May 9-11, 2011 [22].

This study aimed to explore the perspectives of patients undergoing leukemia treatment where most of the patients participated in a local biobanking project. The survey had nine questions containing multiple parts with either fixed choices or 4 or 5-point Likert scale responses. A copy of the questionnaire is available online as Additional file 1.

Recruitment of patients and the collection of responses for the initial and follow-up surveys were done in Toronto, and subsequent analysis was performed by researchers affiliated with the University of Alberta. No patient identifying information was sent to researchers at the University of Alberta. Comparisons between the initial and follow-up surveys were done only for those participants that completed both surveys.

### Statistical analysis

Data were tabulated and analyzed using IBM SPSS 19 for Windows. To determine statistical relevance, Pearson’s Chi-Square (χ^2^) Tests were performed as we aimed to determine whether our observed sample of nominal scale data conformed to an expected distribution. Statistical significance was determined where the null-hypothesis (no difference in categories) was rejected with a *p* < 0.05. In order to maintain statistical accuracy, responses where participants chose more than one response or failed to respond were not included in the final count when performing statistical analysis.

As only 45 of the initial 100 patients completed the follow-up survey we wanted to determine whether there was a potential bias due to non-response. We compared responses between those who completed the follow-up survey with those who did not. Chi-Square tests did not yield any significant differences in the responses of these two groups, and therefore, we do not suspect a response bias.

## Results

### Consent

We first set out to determine patients’ preferences on informed consent for biobanking, asking them to choose one of three consent models: (a) specific/re-consent, (b) one-time general consent, and (c) tiered consent. From our initial set of survey responses, we found that out of 89 patients that responded, 59.6% preferred one-time consent while 30.3% preferred a tiered approach, and 10.1% preferred to re-consent for every future research project (χ^2^ = 32.989, df = 2, *p* < 0.001) (Table 1). Of the 45 participants that completed both initial and follow-up surveys, a significant number of responses changed (n = 20), such that slightly more participants preferred one-time consent and fewer preferred tiered consent (χ^2^ = 47.356, df = 3, *p* < 0.001) (Table 1).

**Table 1:** Patient preferences of different informed consent approaches for biobanking

**Type of consent**	**Initial survey responses**		**Follow-up survey responses**			
	**(n = 89)**	**%**	**Initial (n = 43)**	**%**	**Follow-up (n = 43)**	**%**
**Re-consent:** The researchers should ask you for your permission to use them every time they would like to do a new study	9	10.1	2	9.3	5	11.6
**One time consent:** The researchers should only ask for your permission to use them once, thus allowing researchers to use them for as many studies as they would like	53	59.6	29	67.4	31	72.1
**Tiered consent:** The researchers should provide you with options regarding the types of studies your stored samples and health information could and could not be used in	27	30.3	10	23.3	7	16.3

Due to rounding, totals may not equal 100%.

**Figure 1:**
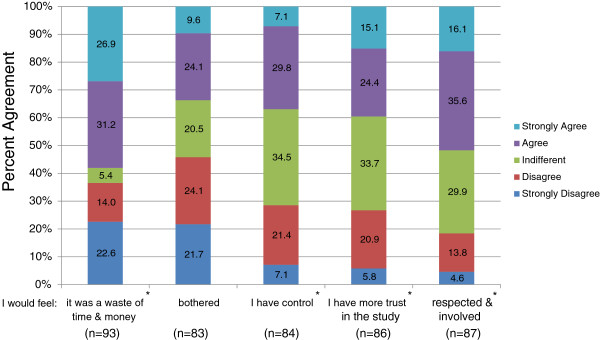
**Patient perspectives on re-consenting for each new study.** Likert scale responses of leukemia patients’ perceptions on re-consent was quantified. Question: How would you feel if you had to give permission to use your stored biological sample and health information before each new study? (a) I would feel it was a waste of time and money. (b) I would feel bothered. (c) I would feel I have control. (d) I would feel more trust in the study. (e) I would feel respected and involved. * indicates the questions to which patient responses were statistically significant. Due to rounding, totals may not equal 100%.

To further understand patient views on informed consent, we asked participants about their views specific to re-consenting. Approximately 58% of patients agreed or strongly agreed that it was a waste of time and money while 36.6% disagreed or strongly disagreed (χ^2^ = 22.108, df = 4, *p* < 0.001) (Figure 1). Patients had a range of opinions when asked whether they would feel bothered by re-consenting with differences not being statistically significant (χ^2^ = 5.976, df = 4, *p* = 0.201). When patients were asked if they would have more control if asked to re-consent, about 21.4% disagreed, 30% agreed, and 34.5% were indifferent (Figure 1). Only 7.1% either strongly agreed or strongly disagreed indicating that only a few patients had strong views on the topic of control (χ^2^ = 26.833, df = 4, *p* < 0.001). Approximately 40% of patients reported that they agreed or strongly agreed on having greater trust in a study if they were allowed to re-consent, 33.7% were indifferent, while 26.7% disagreed or strongly disagreed (χ^2^ = 18.651, df = 4, *p* = 0.001). Lastly, when we asked patients if they would feel respected and involved if they were asked to re-consent, a significant 51.7% agreed or strongly agreed while 18.4% disagreed or strongly disagreed (χ^2^ = 27.540, df = 4, *p* < 0.001) (Figure 1). Follow-up survey responses showed that more patients had less trust in the study (χ^2^ = 44.260, df = 25, *p* = 0.01) and fewer felt respected and involved (χ^2^ = 41.127, df = 25, *p* = 0.022) than what was reported by participants in the initial survey (data not shown).

### Withdrawal

On the topic of withdrawing consent, we found that 62.6% of patients wanted to have the right to withdraw their sample and health information from a biobank (χ^2^ = 6.313, df = 1, *p* = 0.012, n = 99) (Table 2). A particularly interesting finding was that 77.3% of participants changed their response on withdrawal in the follow-up survey (data not shown). This resulted in a significant proportion of patients moving from wanting to have a right to withdraw to deciding that they should not have a right to withdraw consent for future medical research (χ^2^ = 12.259, df = 4, *p* = 0.016) (Table 2).

**Table 2:** Patient perspectives on the right to withdraw informed consent

**Do you think you should have the right to withdraw:**	**Initial survey responses**		**Follow-up survey responses**			
	**(n = 99)**	**%**	**Initial (n = 44)**	**%**	**Follow-up (n = 44)**	**%**
Yes	62	62.6	27	61.4	23	52.3
No	37	37.4	17	38.6	21	47.7

Due to rounding, totals may not equal 100%.

### Anonymity

In order to understand cancer patient perspectives on anonymity, we asked patients whether it was more important to have a sample anonymized (described as no way to trace back to donor) or de-identified (described as researchers do not readily know who the sample belongs to, but there is a way to trace it back to the participant). The results were split: 54.6% of participants desired samples to be anonymized while 45.4% desired de-identification of samples; this difference was not statistically significant (χ^2^ = 0.835, df = 1, *p* = 0.361, n = 97). The follow-up survey showed an increase in the number of participants desiring samples to be anonymized instead of de-identified, but these changes were not statistically significant (χ^2^ = 3.85, df = 2, *p* = 0.146).

### Return of results

Patients were presented with a scenario in which researchers discovered incidental findings related to their health, but unrelated to the initial study. They were asked how researchers should handle this situation: do nothing, inform them, inform their doctor, or inform both them and their doctor. Of the 98 participants that provided responses, only 2% indicated that nothing should be done and 8.2% preferred to know themselves (Table 3). Many patients (22.4%) preferred instead that researchers should notify their physician while most of the patients (67.3%) preferred that researchers should inform both them and their doctor (χ^2^ = 102.327, df = 3, *p* < 0.001).

**Table 3:** Patient preferences on the return of incidental findings

**What do you think researchers should do upon discovering health information?**	**Initial survey responses**	
	**(n = 98)**	**%**
Nothing	2	2.0
Tell me	8	8.2
Tell my doctor	22	22.4
Tell both myself and my doctor	66	67.3

Due to rounding, totals may not equal 100%.

### Public health surveillance

**Figure 2:**
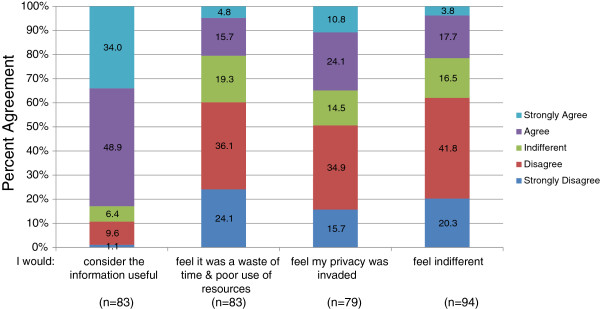
**Patient perspectives on public health surveillance.** Likert scale response of leukemia patients’ perceptions on a factious scenario where a public healthcare worker identifies and notifies them about potential health risks. Question: How would you feel if a policy put a system in place that allowed this public healthcare worker to mail this information to you in order to warn you of your high risk health status? (a) I would consider the information useful. (b) I would feel it was a waste of time and poor use of resources of our public health care dollars. (c) I would feel my privacy was invaded. (d) I would feel indifferent. Due to rounding, totals may not equal 100%.

We asked participants how they would feel if a policy was put in place that allowed a public health worker to analyze their health information and mail information to them related to potential chronic health risks (e.g., an increased risk of diabetes). We found that the majority (82.9%) agreed or strongly agreed that the information would be useful (χ^2^ = 79.298, df = 4, *p* < 0.001) while only 20.5% agreed or strongly agreed it would be a waste of time and a poor use of resources (χ^2^ = 21.880, df = 4, *p* < 0.001) (Figure 2). More participants (50.6%) disagreed or strongly disagreed that their privacy would be invaded (χ^2^ = 16.578, df = 4, *p* = 0.002) (Figure 2). A significant number of responses changed in the follow-up survey for two areas. Half of the 44 participants who completed the follow-up survey changed their mind resulting in about a 10% decrease of participants who agreed or strongly agreed that the information would be useful (χ^2^ = 51.075, df = 25, *p* = 0.002). Additionally, in the follow-up survey, the majority of participants changed their minds resulting in 10% greater response of disagreeing or strongly disagreeing that returning health results was a waste of time and a poor use of resources (χ^2^ = 61.140, df = 25, *p* < 0.001).

### Ownership and decision-making

To assess perspectives on ownership, we asked participants who they believe owned banked samples: the participant, the researcher conducting the research, the institution where the research is being conducted, or the funder(s) of the research. Of the 98 responses received in the initial survey, the majority of participants (62.2%) stated that the research institution owned the samples. But a significant minority reported that research participants (19.4%) or the researcher (16.3%) owned the samples. Only 2% explained that the funder owned the samples (χ^2^ = 79.224, df = 3, *p* < 0.001).

On a similar line of questioning, we asked patients if they had a continuing right to decide what is done with their samples. Of the 95 responses collected in the initial survey, 55.8% indicated they should have the right to decide what is done with their samples (χ^2^ = 1.274, df = 15, *p* < 0.259).

### Trust

**Figure 3:**
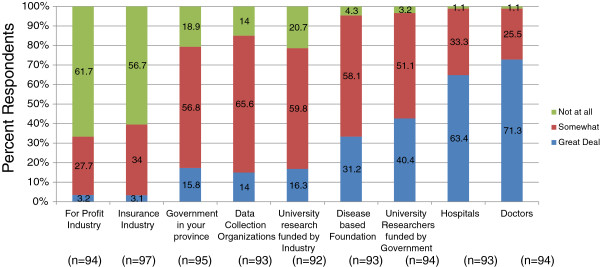
**Patient perspectives on trust in actors and institutions involved in biobanking.** Likert scale response of leukemia patients’ perceptions on their level of trust for different individuals and organizations with the care and use of confidential health information. Question: How much do you trust the following individuals, organizations or groups with the care and use of your confidential health information? (a) For-profit industry, for example, a drug company. (b) Insurance industry. (c) Government in your province. (d) Data collection organizations (Stats Canada/Canadian Institute for Health Information). (e) University research funded by industry. (f) Disease based foundation (e.g., Kidney Foundation, Heart & Stroke). (g) University researchers funded by government. (h) Hospitals. (i) Doctors. Due to rounding, totals may not equal 100%.

The final question of the survey asked patients about their levels of trust in different actors and their organizations in regards to the care and use of their confidential health information. Patients were able to indicate whether they trusted different individuals or organizations “A great deal,” “Somewhat,” or “Not at all,” or they were able to respond as “Don’t know.” Patients had a great deal of trust in doctors (71.3%) (χ^2^ = 73.196, df = 2, *p* < 0.001), hospitals (63.4%) (χ^2^ = 55.473, df = 2, *p* < 0.001), university researchers funded by government (40.4%) (χ^2^ = 37.640, df = 2, *p* < 0.001), and disease based foundations (31.2%) (χ^2^ = 43.103, df = 2, *p* < 0.001) (Figure 3). Patients somewhat trusted the government in their province (56.8%) (χ^2^ = 37.640, df = 2, *p* < 0.001), data collection agencies, e.g., Statistics Canada or the Canadian Institute for Health Information (65.6%) (χ^2^ = 52.966, df = 2, *p* < 0.001), and industry-funded university research (59.8%) (χ^2^ = 32.719, df = 2, *p* < 0.001) (Figure 3). Most patients had quite low levels of trust towards for-profit industry (61.7%) (χ^2^ = 52.621, df = 2, *p* < 0.001) and the insurance industry (56.7%) (χ^2^ = 44.923, df = 2, *p* < 0.001). Of those patients who completed the follow-up survey, 16 participant responses changed such that there was even less trust towards for-profit industry (χ^2^ = 27.856, df = 12, *p* = 0.006), and 15 participants changed responses showing slightly less trust towards hospitals (χ^2^ = 7.979, df = 3, *p* = 0.046) (data not shown).

## Discussion

Much public perception research on the ethical and legal aspects of research involving biobanks has been performed with patient populations, including adults and children, and healthy volunteers (e.g., general public, US veterans, and racial groups) in many jurisdictions. Yet little research has been conducted to specifically assess the perceptions of Canadian cancer patients. Surveying Canadian cancer patient perceptions provides an opportunity to compare these perceptions to those of different populations surveyed in other research studies. This work helps to provide a picture of the breadth of opinions relevant to the ethical and legal controversies associated with biobanking.

### Consent

The topic of informed consent as it relates to biobanking has dominated public perception studies. A recent review shows that 51% of the 87 different empirical studies on biobanking discuss stakeholder perceptions on consent [23]. In our study, we limited our question to three consent types, despite the multitude of possible consent models for biobanking, so as to maintain simplicity in our questionnaire and because many of the ethical and legal issues for other variations of consent are similar [24]. Our results clearly showed that of the three options provided (re-consent, one-time consent, and tiered consent), the majority of patients (59.6%) preferred a one-time consent. However, many preferred a form of consent that was more specific and ongoing in nature (e.g., some preferred to re-consent and nearly a third chose tiered consent). Our results corroborate findings from some studies done in other jurisdictions. One study analyzing consent preferences from breast cancer survivors in the Netherlands showed that 61% indicated that they would like to choose the type of future research, 56% preferred a one-time general consent model, and 45% indicated that they would rather give permission for the current research, but not for other future research [25]. Another study examined the views on consent of African American and White American cancer patient groups who attended two healthcare facilities in Atlanta, Georgia. The researchers found that although the vast majority of patients from both cohorts were willing to participate (95%), 57% desired that researchers choose the type of future research using their samples while 10% wanted to choose using a checklist of options, and 33% explained either option would be fine [26]. It is unknown why there are differences between different populations of cancer patients, but many factors could influence the difference in opinions including the ways in which the questions were posed in the surveys.

Results of studies with the general population (who may or may not be patients) are in some respects comparable, but interesting differences are also present. In contrast to our study, a US nationwide study that offered participants different choices showed that 48% preferred blanket consent (consent for any future research), 42% preferred being asked at the initiation of each study, and 10% preferred tiered consent [27]. In addition, a study conducted by our group surveying 1201 Albertans demonstrated that 51.8% preferred one-time consent, 30.2% chose tiered consent, and 18.0% preferred re-consent [22]. The slightly higher preference for a one-time consent by patients as compared to those in the general public may be due to their personal interest in the research and the high degree of trust in their medical team. In total, these results are similar to findings from our study and indicate that while the majority of participants are comfortable with a one-time consent, many prefer tiered consent or re-consent approaches indicating a desire for control.

We asked patients additional questions on re-consent and compared our results to a survey of 1201 Albertans [22], and a nationwide study of approximately 8735 Americans [27] (Table 4). Approximately 58% of patients and Albertans reported that re-consent was a waste of time and money compared to only 27% of Americans. Interestingly, 33.7% of patients and 26% of Americans thought it would be bothersome to re-consent in contrast to 51.9% of Albertans who felt this way. On other points, it seemed overall that cancer patient opinions from our survey on control, trust, or feeling respected and involved differed from the US general public and aligned more with the views of Albertans (Table 4). One possible explanation is that perhaps cancer patients, a group that has frequent interaction with the healthcare system, have a higher degree of trust in their physicians and researchers than the general public. Their situation may also mean that they view the relevant research as important and, as a result, feel less need to provide specific and ongoing consent.

**Table 4:** A comparison of opinions on re-consenting for future biobanking research

	**Patient survey (%)**		**US population (%)**		**Albertans (%)**	
**I would feel:**	**Agree-strongly agree**	**Disagree-strongly disagree**	**Agree-strongly agree**	**Disagree-strongly disagree**	**Agree-strongly agree**	**Disagree-strongly disagree**
It was a waste of time & money	58.1	36.6	27	73	58.5	30.8
Bothered	33.7	45.8	26	74	51.9	39.1
I have control	36.9	28.5	75	25	51.0	32.5
More trust in the study	39.5	26.7	75	25	48.1	34.8
Respected and involved	51.7	18.4	81	19	56.7	26.5

Due to rounding, totals may not equal 100%.

### Withdrawal

Very little public perception research covers the topic of withdrawing consent in the context of biobanking [23] and in many cases questions surrounding withdrawal are intimately tied to issues of privacy, consent, and trust. Most of the patients in our study (62.6%) wanted to have the right to withdraw consent at any time. Our results found less support for unlimited withdrawal as compared to Albertans, where 71.2% indicated that participants should have a right to withdraw [22]. However, our results were similar to a Swedish study where 62% of participants reported feeling positive about the right to withdraw [28]. Yet there are studies that have found very different results. For example, an evaluation of 600 adult Egyptian patients found that only 28.8% believed in the right to withdraw their blood samples [29]. There is also evidence to suggest that some participants do not perceive a reason to withdraw, but feel it is an option that should be maintained in case others had concerns about the study [30]. Reasons for withdrawal could include a breach of security or scandal, negative news media about the research, changes to a participant’s health status, burdensome requests from researchers, and negative responses from peers [31]. Most interesting was that compared to the initial survey results, more patients indicated that they should not have the right to withdraw in the follow-up survey. We speculate that this observation may be partially explained by the high degree of trust patients place in university-funded researchers and medical staff, and that they believe that biobanking research could advance medical treatments for leukemia.

The right to withdraw from research is supported by virtually all research ethics policies, but there are issues with the practicality of withdrawing consent as it relates to biobanking. Some are concerned that the right to withdraw consent for biobanking may be eroding because it could cause sample bias in a given population [32-34]. Yet withdrawing consent is a fundamental research ethics norm [35]. Overall, results from our study showed that patients reported they should have the right to withdraw consent at any time. An additional complication in the context of banking samples from cancer patients is determining who has the authority to withdraw samples when there is no legal will or declaration of a proxy. This is especially important in the case of collecting and storing samples from cancer patients because patients could die soon after donation. Additional empirical research examining the complexities of the right to withdraw and the perceptions of different stakeholders is needed.

### Anonymity

Patient preferences of whether samples are either deidentified or fully anonymized and cannot be traced back to the donor were roughly 50–50. Yet having samples fully anonymized becomes problematic when collecting and storing samples involving cancer patients because the diagnosis of samples is linked to the tailoring of treatment regimens for individual patients. It is unclear from our questionnaire whether patients understood this caveat when responding to the survey question.

### Return of results

In general, research tells us that the general public and patients have a strong desire to receive individual results either for them or their descendants [23]. Our results confirm this trend. Indeed, about 98% of those surveyed wanted this information to be disclosed to themselves, their physicians, or both. This is, in fact, one of the few areas of almost universal agreement.

These results are similar to the perceptions of Albertans where 90% of participants wanted to be informed of research results in some way [22]. In general, many studies confirm that the public and patients have a strong preference to know research results [29,36-38]. However upon further examination of the types of research results participants desire, the numbers differ. Several studies indicate that many participants want to receive results in situations where treatment options are available and have a lower desire to know results for either untreatable conditions or where the significance of the finding is not well understood [39-41]. Our results clearly show that cancer patients have a high desire to know the results of research. Yet we reason that if patients were provided details on the different types of results that might be returned, their views are likely to be nuanced as we have seen in other studies.

### Ownership and decision making

In the few surveys that capture perceptions on ownership, about 23-53% of participants report that the participant themselves are the owners of the samples [25,41,42]. Surprisingly, our survey results showed that only 19.4% of patients believed they were the owners and the majority (62.2%) believed that research institutions owned the samples. The survey of Albertans also showed a relatively high number of participants (44.3%) believed that the research institution owned samples, although a significant minority (25.7%) of respondents believed they owned the samples [22]. We speculate that the high number of patients believing that the research institution owns the samples could be because they perceive government-funded researchers to be trustworthy and, therefore, feel comfortable with the research institution having an ownership-like interest. In addition, as we did not define the term “research institution,” patients may conflate research institution with the hospital where they were treated (which, admittedly, is often the case). This explanation is supported by another study that observed that many patients responded that the hospital owned the samples [42]. Yet despite many patients believing that research institutions own samples, the majority of patients (55.8%) indicated that they have the right to decide what is done with their samples. These findings are reinforced from a study showing that many participants prefer multiple data sharing options and want some control on decision-making [43]. More research is needed to better understand participant conceptions of ownership, the right to decide, and the factors that influence desires for control over their samples and health information.

### Trust

Patients reported higher levels of trust in doctors, hospitals, and government-funded university researchers, moderate levels of trust in their provincial government, and low levels of trust in for-profit industry and insurance companies. Near identical results were also seen when surveying Albertans [22] and similar findings were seen in other public perception research [44,45]. Other than trusting actors in various organizations, there is some data suggesting that participants show less trust of biorepository research that has the potential to cause stigmatization or discrimination, or goes against deeply held cultural or religious beliefs, e.g., lineage determination, behavioral disorders, and inbreeding [5,10,20,36,44,45]. Although our survey questions did not discuss the different areas in which research samples may be used, in general, cancer patients have similar high levels of trust in the organizations as seen in other studies [22].

### Limitations

The survey has several important limitations that merit discussion. It considers the opinions of leukemia patients from a specific geographical location, and opinions of cancer patients from other areas or other types of patients (i.e., surgical) may vary. To maintain anonymity, our survey did not gather information related to age, education, ethnicity, or religious background or belief system, and correlate this information with participant responses. One limitation specific to our survey was that some participants did not complete questions containing multiple parts (e.g., A through E) where each part required a single Likert scale response. We suspect that this may be due to misinterpreting the instructions which asked for a single response to each part, and this may bias responses as these were excluded from the analysis. As our survey was meant to capture broad patient perspectives and compare them with those of other Canadian stakeholders, we did not pose questions that attempted to determine the reasons behind participant responses. Future qualitative research with cancer patients would complement our survey and permit a richer understanding of reasons behind differing viewpoints.

## Conclusions

Our survey results of Canadian cancer patients’ perceptions on the ethical and legal issues of biobanking showed several interesting differences and similarities with the results of other surveys. Results on consent showed that many patients desire some form of control. As with most other studies, there seems to be general agreement that returning results of research and allowing participants to withdraw consent are desirable practices. It is interesting to note that as compared to the results of other surveys, more patients in our study felt that they should not have the right to withdraw consent. Although a statistically relevant association was not found, we speculate that because this patient population has a high degree of trust in their doctors, the hospital, and university-based researchers, they may not feel as strongly about a need for a right to withdraw. In addition, their belief in the importance of biobanking research for leukemia may also influence their views. Similarly, the high numbers of patients believing that the research institution owns their samples could also be explained, in part, by the elevated levels of trust they have for their clinical staff and researchers. Our study is one of a few that examines Canadian patient perceptions on biobanking and provides an important contribution to the broader understanding and public discourse surrounding biobanking, and thus has relevance for the development of research policy on biobanking.

## Competing interests

The authors declare they have no competing interests.

## Authors’ contributions

All authors made substantial contributions to the conception and design of the research study, including drafting survey questions. ZM analyzed and interpreted the data, prepared tables and figures, and initially drafted the manuscript. JC disseminated the survey to participants, collected survey responses and critically revised the manuscript for important intellectual content. CR helped analyze and interpret the data, performed the statistical analysis, and revised the manuscript for important intellectual content. MM and JW helped analyze and interpret the data, and critically revised the manuscript for important intellectual content. TC helped write the initial draft of the manuscript, helped analyze and interpret the data, and critically revised the manuscript for important intellectual content. All authors have given final approval of the manuscript to be published.

## Pre-publication history

The pre-publication history for this paper can be accessed here:

http://www.biomedcentral.com/1755-8794/6/8/prepub

## Supplementary Material

Additional file 1 **Stakeholder survey: perspectives on biobanking and tissue sampling.** General population/patient population questionnaire. Click here for file
