# Supplementary material for: Cancer patient perceptions on the ethical and legal issues related to biobanking
Source: BMC Med Genomics. 2013 Mar 8;6:8. doi: 10.1186/1755-8794-6-8 (PMC3599691; doi:10.1186/1755-8794-6-8)
Supplement: Additional file 1 — Stakeholder survey: perspectives on biobanking and tissue sampling. General population/patient population questionnaire. [file 1755-8794-6-8-S1.pdf]

Stakeholder Survey: Perspectives on Biobanking and Tissue Sampling

General Population/Patient Population Questionnaire

1) As you know for lots of different health studies researchers collect biological samples, such as blood, tissue and urine, and health information, such as family medical history and genetic information. When these are collected, usually with consent, they are often stored for future medical research, which may not be known at the time of a collection.

   Given this, imagine a scenario where researchers have collected and stored your biological samples and health information. When it came time for them to be used for research, do you think: ***(Choose only one answer):***

   a) The researchers should ask you for your permission to use them every time they would like to do a new study _____
   b) The researchers should only ask for your permission to use them once, thus allowing researchers to use them for as many studies as they would like _____
   c) The researchers should provide you with options regarding the types of studies your stored samples and health information could and could not be used in. _____

2) How would you feel if you had to give permission to use your stored biological sample and health information before each new research study: ***(Answer a through e with one of 5 options to choose from.)***

   a) I would feel that it was a waste of time and money
      Strongly Disagree __ Disagree __ Indifferent __ Agree__ Strongly Agree__
   b) I would feel bothered
      Strongly Disagree __ Disagree __ Indifferent __ Agree__ Strongly Agree__
   c) I would feel I have control
      Strongly Disagree __ Disagree __ Indifferent __ Agree__ Strongly Agree__
   d) I would have more trust in the study
      Strongly Disagree __ Disagree __ Indifferent __ Agree__ Strongly Agree__
   e) I would feel respected and involved
      Strongly Disagree __ Disagree __ Indifferent __ Agree__ Strongly Agree__

3) In a scenario where researchers had collected and stored biological samples (such as blood, tissue, urine and genetic information) and health information from you for future medical research, do you think you should have the right to withdraw your consent and sample at any time? ***(Choose only one answer)***
   a) Yes ____
   b) No ____

4) Suppose biological samples can be completely anonymized or de-identified. An anonymized sample is when there is no way to trace it back to the donor. A de-identified sample is when researchers do not readily know who the sample belongs to, but there is a person, or a few people, that can trace the sample back to the research participant. Anonymized samples are thought to best protect a research participant's privacy, but once a sample is anonymized it is impossible for him or her to withdraw their sample.

   In your opinion, what do you feel is more important: ***(Choose only one answer)***
   a) Having a sample anonymized _____
   b) Having a sample de-identified, providing the right to withdraw from the study _____

5) Imagine a scenario where a researcher does some tests on your sample and finds out information related to your health that is unrelated to the study for which you had originally provided your sample for. For example, consider the researcher discovered that you were at an increased risk for cancer or heart disease, or carry a gene that indicated a predisposition to depression.

   What do you think researchers should do upon discovering this information? ***(Choose only one answer)***

   a) Nothing ____
   b) Tell me _____
   c) Tell my doctor _____
   d) Tell both myself and my doctor ______

6) Imagine a scenario where a public healthcare worker could access your health information without your knowledge and identify if you were at risk for a chronic health condition, including obesity, diabetes, hypertension, or chronic kidney disease. How would you feel if a policy put a system in place that allowed this public healthcare worker to mail this information to you in order to warn you of your high risk health status. ***(Answer a to d with one of the 5 options to choose from)***

   a) I would feel that it was a waste of time and poor use of our public health care dollars.
   Strongly Disagree __ Disagree __ Indifferent __ Agree__ Strongly Agree__
   b) I would be feel that my privacy was invaded
   Strongly Disagree __ Disagree __ Indifferent __ Agree__ Strongly Agree__
   c) I would feel indifferent
   Strongly Disagree __ Disagree __ Indifferent __ Agree__ Strongly Agree__
   d) I would consider the information useful
   Strongly Disagree __ Disagree __ Indifferent __ Agree__ Strongly Agree__

7) For various types of health research, biological samples (such as, blood, tissue, urine and genetic information) are often stored and collected. If you provided a sample that was part of such a collection, who do you think owns these samples? ***(Choose only one answer.)***
   a) You __
   b) The researcher conducting the research ___
   c) The institution where the research is being conducted ___
   d) The funder (or funders) of the research if different from above ___

8) For various types of health research, biological samples (such as, blood, tissue, urine and genetic information) are often stored and collected. If you provided a sample that was part of such a collection, this sample would contain your genetic information. Given this, do you think you have the continuing right to decide on what is done with your sample? ***(Choose only one answer)***

a) Yes ___
b) No ____

9) How much do you trust the following individuals, organizations or groups with the care and use your confidential health information? ***(Answer a to i with one of the 4 options to choose from.)***

a. Insurance industry __
A great deal ___ Somewhat ___ Not at all ____ Don't Know ___
b. Government in your province __
A great deal ___ Somewhat ___ Not at all ____ Don't Know ___
c. For profit Industry, for example, a drug company
A great deal ___ Somewhat ___ Not at all ____ Don't Know ___
d. Data collection organizations (Stats Canada/Canadian Institute for Health Information) __
A great deal ___ Somewhat ___ Not at all ____ Don't Know ___
e. Disease based foundation (eg. Kidney Foundation, Heart & Stroke) __
A great deal ___ Somewhat ___ Not at all ____ Don't Know ___
f. University researchers funded by government ___
A great deal ___ Somewhat ___ Not at all ____ Don't Know ___
g. University research funded by industry
A great deal ___ Somewhat ___ Not at all ____ Don't Know ___
h. Hospitals __
A great deal ___ Somewhat ___ Not at all ____ Don't Know ___
i. Doctors ___
A great deal ___ Somewhat ___ Not at all ____ Don't Know ___
